# Commentary on ‘Surface markers associated with chondrogenic potential of human mesenchymal stromal/stem cells’

**DOI:** 10.12688/f1000research.21207.1

**Published:** 2020-01-23

**Authors:** Zhihua Lu, Lianqi Yan, Ming Pei

**Affiliations:** 1 Stem Cell and Tissue Engineering Laboratory, Department of Orthopaedics, West Virginia University, Morgantown, WV, 26506, USA; 2 Department of Orthopaedics, Orthopaedics Institute, Clinical Medical College of Yangzhou University, Subei People’s Hospital of Jiangsu Province, Yangzhou, Jiangsu, 225001, China; 3 WVU Cancer Institute, Robert C. Byrd Health Sciences Center, West Virginia University, Morgantown, WV, 26506, USA

**Keywords:** mesenchymal stromal/stem cell, surface marker, proliferation, chondrogenic differentiation, cartilage

## Abstract

In the last decade, researchers have searched for predictive surface markers of multipotent mesenchymal stromal/stem cells (MSCs) for ensuring improved therapeutic outcomes following cartilage damage in humans. However, we have achieved only limited progress because of the challenge presented by conflicting data. This commentary provides some evidence to prove a lack of success with current efforts, including an inconsistency in accepted surface markers and chondrogenic potential of MSCs as well as the tissue source–dependent MSC surface markers that correlate with chondrogenic potential. A brief discussion on these disputed topics and perspective about functionally predictive surface markers and standardization of analytic procedures are also highlighted.

## Introduction

As a leading cause of disability among adults, osteoarthritis often results from a biochemical breakdown of articular cartilage in joints ^1^. Articular cartilage has a poor intrinsic healing capacity because of its avascular structure, immobility of chondrocytes, and low mitotic activity. Compared with conventional surgical methods, autologous cell therapy, growth factor therapy, and biomaterials provide more promising approaches for clinical treatment ^2^. Human multipotent mesenchymal stromal/stem cell (MSC)-based cell therapy is expected to deliver a promising treatment for cartilage repair because of easy isolation of cells from mesenchymal tissues with higher proliferative and chondrogenic potential ^3, 4^. Given that MSCs exist in a number of tissues and organs, such as bone marrow, synovial membrane, and adipose tissue ^5– 7^, to compare research outcomes and promote the development of MSC-based therapy, the International Society for Cellular Therapy defined human MSCs in 2006 ^8^. First, *in vitro* culture of MSCs must have the ability to adhere to plastic substrates; second, MSCs should express cluster of differentiation 73 (CD73), CD90, and CD105 (>95%), which are measured by flow cytometry. Meanwhile, CD14, CD19, CD34, CD45, and HLA class II should be negative (≤2% positive). Third, MSCs must have osteogenic, chondrogenic, and adipogenic capacities *in vitro*.

**Figure 1.**
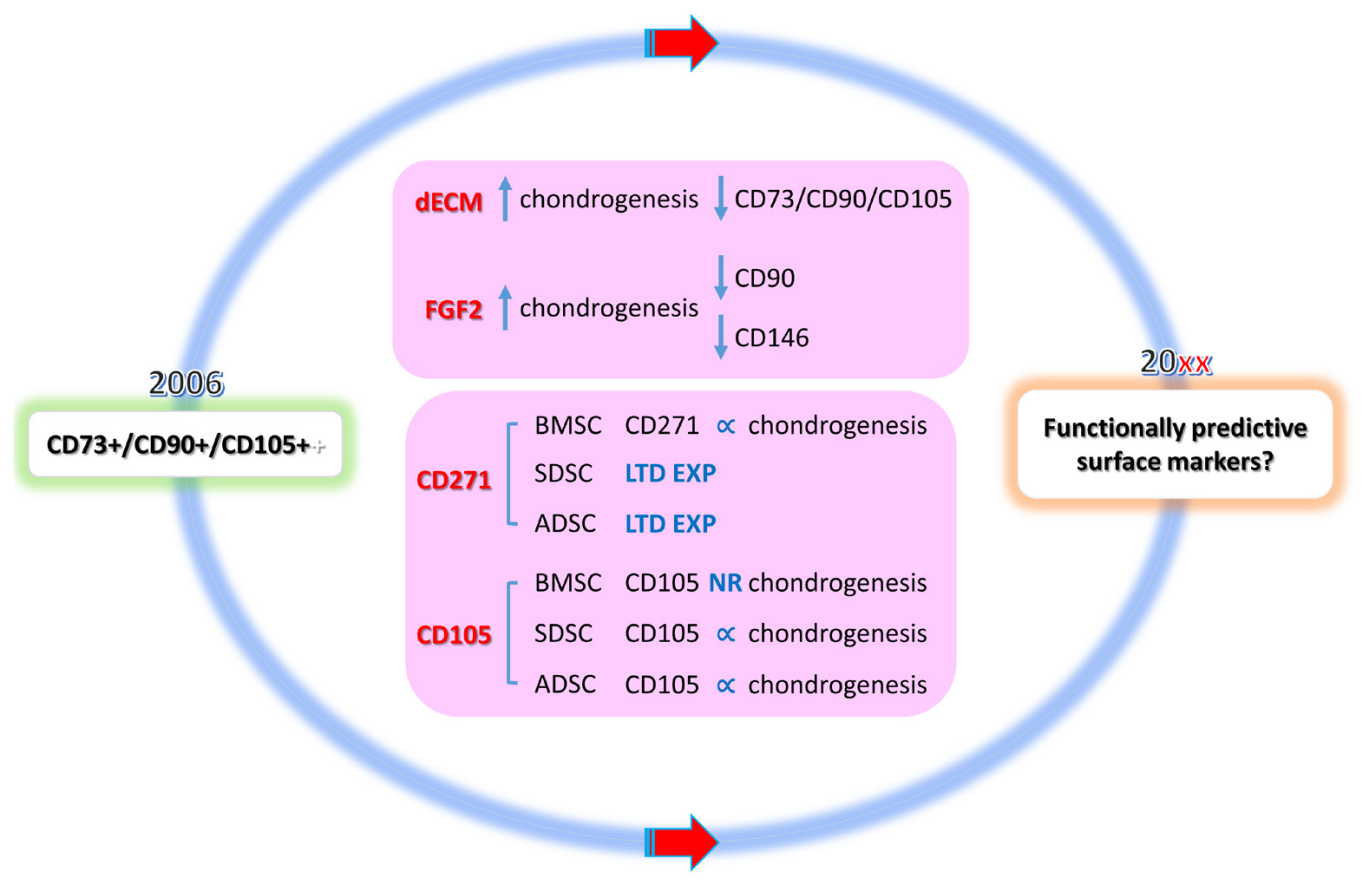
Schematic diagram of our Commentary on ‘Surface markers associated with chondrogenic potential of human mesenchymal stromal/stem cells’. Human mesenchymal stromal/stem cells (MSCs) were defined in 2006 by the International Society for Cellular Therapy. Unfortunately, currently accepted predictive surface markers do not seem to be ideal candidates to predict MSCs with chondrogenic potential in terms of inconsistency of currently accepted surface markers and chondrogenic potential of MSCs and tissue source–dependent MSC surface markers that correlate with chondrogenic potential. ** Are functionally predictive surface markers the next target for sorting MSCs in cartilage engineering and regeneration? ∝, positive correlation; ADSC, adipose-derived stromal/stem cell; BMSC, bone marrow-derived stromal/stem cell; dECM, decellularized extracellular matrix; FGF2, fibroblast growth factor 2; LTD EXP, limited expression; NR, not related; SDSC, synovium-derived stromal/stem cell.

Increasing evidence has shown that human MSC subpopulations which were sorted by some surface markers had better chondrogenic potential for cartilage regeneration. Researchers are trying to find predictive MSC surface markers for ensuring improved therapeutic outcomes. Although some promising MSC surface markers have been comprehensively reviewed ^9– 11^, Alegre-Aguarón *et al*. questioned the correlation between stem cell surface markers and chondrogenic potential ^12^. This commentary provides some evidence to prove a lack of success with current efforts, including an inconsistency in accepted surface markers and chondrogenic potential of MSCs as well as the tissue source–dependent MSC surface markers that correlate with chondrogenic potential. A brief discussion on these disputed topics and perspective about functionally predictive surface markers and standardization of analytic procedures are also highlighted ( Figure 1).

## Inconsistency of currently accepted surface markers and chondrogenic potential of MSCs

Increasing evidence suggests that environmental preconditioning revitalizes the proliferation and chondrogenic capacity of adult stromal/stem cells ^13^. Given two good examples that decellularized extracellular matrix (dECM) expansion or fibroblast growth factor 2 (FGF2) pretreatment can promote human MSC proliferation and chondrogenic potential, there is an inconsistency in accepted surface markers and chondrogenic potential of MSCs.

### dECM expansion

Recent reports demonstrated that dECM deposited by synovium-derived stromal/stem cells (SDSCs) provided an *in vitro* microenvironment for SDSC expansion, which dramatically improved proliferation and enhanced chondrogenic potential ^14– 16^. The flow cytometry data reported by Li *et al*. showed that, compared with surface markers of human SDSCs grown on tissue culture plastic (TCP), the percentage of CD29, CD90, and CD105 expression of SDSCs grown on dECM decreased slightly but the median fluorescence intensity (MFI) declined dramatically; interestingly, both the percentage and MFI of stage-specific embryonic antigen 4 (SSEA4) increased ^17^. Zhang *et al*. also found that, despite nearly 100% expression in SDSCs after expansion on either TCP or dECM substrates, CD29, CD90, and CD105 declined dramatically at the MFI in dECM-expanded SDSCs; the MFI of SSEA4 in dECM-expanded cells increased slightly while the percentage doubled ^18^. Interestingly, real-time quantitative polymerase chain reaction results showed that SRY-Box9 ( *SOX9*), type II collagen ( *COL2A1*), and aggrecan ( *ACAN*) were significantly upregulated during chondrogenic induction in SDSCs from the dECM group ^18^. In those two studies, SSEA4 was found to be the only surface marker under evaluation that increased when dECM improved the chondrogenic potential of human SDSCs. However, Li *et al*. showed that SSEA4(+) expression did not favor human SDSC chondrogenesis because enhanced chondrogenesis occurred in the SSEA4(−) population of cells ^19^.

### FGF2 pretreatment

In 2011, Kim *et al*. found that the expression percentage of surface marker CD49a in human SDSCs decreased with pretreatment using FGF2 and that FGF2 exerted no effect on the expression levels in CD29, CD44, CD73, CD105, and CD166 ^20^. In that study, the size, weight, and glycosaminoglycan (GAG) accumulation of pellets increased following FGF2 supplementation during cell expansion. In another study, after seven days of monolayer expansion in the presence of FGF2, human SDSCs became significantly smaller and showed a fibroblast-like appearance as well as a decrease in MFI for CD29, CD90, and CD10; however, FGF2-pretreated SDSCs showed significantly increased chondrogenic potential ^21^. Hagmann *et al*. reported that FGF2 pretreatment suppressed CD146 expression in human bone marrow–derived stromal/stem cells (BMSCs) and promoted chondrogenic differentiation ^22^. These studies demonstrated that, during *ex vivo* expansion, FGF2 is an effective agent to promote human MSC proliferation and chondrogenic potential via upregulation of SOX9 ^23^. However, measured surface markers did not show a positive correlation with the proliferative and chondrogenic potential of MSCs.

## Tissue source–dependent MSC surface markers that correlate chondrogenic potential

Some surface markers associated with chondrogenic potential are not equally expressed in all tissue-specific stromal/stem cells. The evidence shown in this section supports the conclusion that the predictive capacity of CD271 and CD105 for MSC chondrogenic potential is tissue source–dependent in terms of MSCs from synovium, bone marrow, and adipose.

### CD271

CD271, a low-affinity nerve growth factor receptor, is considered to be a highly selective surface marker for BMSCs ^24^. The reports from Mifune *et al*. ^25^ and Calabrese *et al*. ^26^ showed that CD271(+) BMSCs from freshly isolated cells had higher chondrogenic potential as evidenced by increased expression of chondrogenic genes in pellet culture with induction medium compared with CD271(−) BMSCs. Petters *et al*. demonstrated that, without *ex vivo* expansion, human bone marrow–derived CD271(+) mononuclear cells could generate sufficient articular cartilage constructs exhibiting high cell viability and remarkable chondrogenic matrix deposition in a type I collagen hydrogel ^27^.

Given that CD271 plays an important predictive role in chondrogenic potential of human BMSCs, there is still controversy over whether human SDSCs express CD271 ^28, 29^. Some studies reported that CD271 was expressed only in the synovial membrane of patients with osteoarthritis ^29, 30^. Interestingly, increasing evidence indicates that SDSCs are tissue-specific stromal/stem cells for chondrogenesis ^31^ and present superior chondrogenic potential and less hypertrophy compared with BMSCs ^32^.

Despite the low abundance of CD271(+) subpopulation within stromal vascular fraction cells, Quirici *et al*. found that the CD271(+) subpopulation of adipose-derived stromal/stem cells (ADSCs) had high increments in cell proliferation when compared with unsorted ADSCs ^33^. Research by Kohli *et al*. showed that CD271(+) ADSCs from *ex vivo* expansion had a superior ability to promote cartilage repair compared with unsorted ADSCs ^34^. However, the study by Beckenkamp *et al*. showed that, in freshly isolated cells, CD34(+)CD271(+) ADSCs displayed similar *in vitro* chondrogenic potential at passage 3 compared with CD34(+)CD271(−) ADSCs ^35^.

### CD105

CD105 (endoglin) is a transmembrane protein that regulates cellular proliferation, differentiation, and migration ^36^. Cleary *et al*. found that the percentage of CD105 in human BMSCs was not related to subsequent chondrogenic potential since CD105 expression did not change during cell expansion when chondrogenic potential decreased ^37^. Interestingly, this outcome seems to be inconsistent with the role of CD105 in SDSCs and ADSCs. Arufe *et al*. in 2009 ^38^ and Chang *et al*. in 2013 ^39^ demonstrated that the cellular subset of CD105(+) SDSCs from *ex vivo* expansion possessed greater chondrogenic capacity than the CD105(−) SDSC subset. Jiang *et al*. found that CD105(+) ADSC subpopulation in *in vitro* culture had a much stronger chondrogenic potential than CD105(−) subpopulation and had more intensive immunostaining of type II collagen and higher gene expression of *COL2A1* and *ACAN* following chondrogenic induction ^40^. There is also a report that myrtucommulone-A treatment reduced CD105 expression in expanded human ADSCs along with reduced chondrogenic potential ^41^.

## Discussions and perspectives

In this commentary, we discussed that the same surface markers might perform differently in predicting the chondrogenic potential of MSCs isolated from different tissues. We also highlighted the inconsistency in currently accepted surface markers and chondrogenic potential of MSCs, which brings up the challenge to find more reliable surface markers to meet the demands of future regenerative medicine. A 2017 report from Dickinson *et al*. ^42^ raised the concept of “functionally predictive surface markers”, which may convey a promising method to address this issue. In the article, the authors used a genomic profiling strategy to find a functional MSC surface marker that can predict enhanced chondrogenic potential. They found that receptor tyrosine kinase-like orphan receptor 2 (ROR2), the Wnt5a receptor, was upregulated in highly chondrogenic clones and used ROR2 to sort the MSC subpopulation which can produce enhanced cartilage constructs with superior efficacy in an animal cartilage repair model. As a functionally predictive surface marker, ROR2 is believed to be important for chondrogenesis, including initial morphology of the cartilage anlagen and subsequent tuning of mature cartilage ^43^, as well as mediating Wnt5a signaling in enhancing chondrogenesis by activation of *SOX9* ^44^. Intriguingly, a recent report from Stüdle *et al*. did not find human BMSCs to express ROR2 since the percentage of ROR2(+) cells was lower than 0.1% ^45^. The authors also found that high variability both across the donors and across clonally derived strains in BMSCs challenged chondrogenic differentiation outcomes. These results indicate that there is a long way to go to find functionally predictive surface markers for stromal/stem cell–based cartilage engineering and regeneration.

Although many studies have focused on the correlation between human MSC surface markers and chondrogenic potential, there is a lack of standard procedures to quantify surface marker expression. Some procedures might influence the outcome, such as enzyme-dependent cell-detaching methods. A 2017 report compared the effect of cell-detaching methods on the positive proportion of surface markers of cultured SDSCs ^46^. They found that trypsin (catalog number 25200072; Thermo Fisher Scientific, Waltham, WA, USA) obviously reduced the percentage and MFI of CD73(+) cells and CD105(+) cells but had little effect on the percentage of CD90 expression. They also found that TrypLE (catalog number 12563011; Thermo Fisher Scientific) had no influence on the positive proportion of tested surface markers at 30 minutes of digestion but dramatically reduced the CD44(+), CD49c(+), CD73(+), CD140a(+), and CD140b(+) cell populations at 60 minutes of digestion. Collagenase (catalog number C9263; MilliporeSigma, Burlington, MA, USA) was found to reduce the CD58(+), CD105(+), and CD140b(+) cell populations at 120 minutes of digestion. By using flow cytometric analysis, most researchers tend to measure the percentage rather than the MFI of surface markers to assess the influence on MSC chondrogenic potential. However, the percentage analysis method is easily affected by outliers and disregards fluorescent intensity shifts that may show how proliferation and differentiation are progressing by a change in the level of surface marker expression. To overcome the limitations of percentage, Chan *et al*. proposed an analysis method based on MFI because of its robustness against outliers and increased accuracy ^47^. Therefore, the relationship between surface markers and chondrogenic ability should be further studied by a method including the MFI of MSCs.
